# A Narrative Review on C3 Glomerulopathy: A Rare Renal Disease

**DOI:** 10.3390/ijms21020525

**Published:** 2020-01-14

**Authors:** Francesco Paolo Schena, Pasquale Esposito, Michele Rossini

**Affiliations:** 1 Department of Emergency and Organ Transplantation, Renal Unit, University of Bari, 70124 Bari, Italy; michelerossini@libero.it; 2 Schena Foundation, European Center for the Study of Renal Diseases, 70010 Valenzano, Italy; 3 Department of Internal Medicine, Division of Nephrology, Dialysis and Transplantation, University of Genoa and IRCCS Ospedale Policlinico San Martino, 16132 Genova, Italy; p.esposito@smatteo.pv.it

**Keywords:** C3 glomerulopathy, Dense deposits disease, C3 glomerulonephritis, CFHR5 nephropathy

## Abstract

In April 2012, a group of nephrologists organized a consensus conference in Cambridge (UK) on type II membranoproliferative glomerulonephritis and decided to use a new terminology, “C3 glomerulopathy” (C3 GP). Further knowledge on the complement system and on kidney biopsy contributed toward distinguishing this disease into three subgroups: dense deposit disease (DDD), C3 glomerulonephritis (C3 GN), and the CFHR5 nephropathy. The persistent presence of microhematuria with or without light or heavy proteinuria after an infection episode suggests the potential onset of C3 GP. These nephritides are characterized by abnormal activation of the complement alternative pathway, abnormal deposition of C3 in the glomeruli, and progression of renal damage to end-stage kidney disease. The diagnosis is based on studying the complement system, relative genetics, and kidney biopsies. The treatment gap derives from the absence of a robust understanding of their natural outcome. Therefore, a specific treatment for the different types of C3 GP has not been established. Recommendations have been obtained from case series and observational studies because no randomized clinical trials have been conducted. Current treatment is based on corticosteroids and antiproliferative drugs (cyclophosphamide, mycophenolate mofetil), monoclonal antibodies (rituximab) or complement inhibitors (eculizumab). In some cases, it is suggested to include sessions of plasma exchange.

## 1. Introduction

Membranoproliferative or mesangiocapillary glomerulonephritis (MPGN) has been traditionally classified based on the light and electron microscopy (EM) findings; here, there are three categories: type I, characterized by the presence of immune deposits in the subendothelial space and mesangium of glomeruli; type II, characterized by C3 deposits within the mesangium and in the basement membranes highly osmiophilic on electron microscopy (dense deposits disease; DDD); and type III, which is a variant of type I [1].

**Figure 1:**
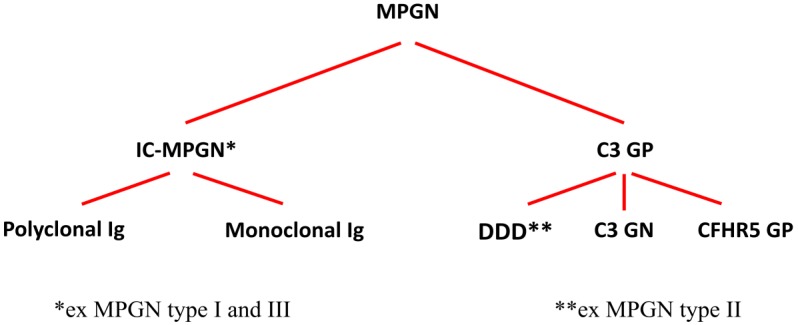
Classification of the membranoproliferative glomerulonephritis (MPGN). Abbreviations: IC-MPGN (immune complex MPGN); Ig (immunoglobulin); C3 GP (C3 glomerulopathy); DDD (dense deposits disease); C3 GN (C3 glomerulonephrits); CFHR5 GP (complement factor H-related protein 5 glomerulopathy).

In August 2012, a group of experts in renal pathology, nephrology, complementology, and complement therapeutics organized a consensus conference on the C3 glomerulopathy (C3 GP), meeting in Cambridge, UK [2]. Subsequently, new information on the complement system has increased our understanding of the MPGN, which has been divided into two groups: (i) MPGN caused by immune complexes (IC-MPGN) that can be caused by polyclonal or monoclonal IgG and (ii) complement-mediated glomerulonephritis (Figure 1).

The term C3 GP includes a group of nephritides based on the abnormal control of the alternative pathway of the complement system that causes a dominant accumulation of C3 fragments in glomeruli, as evidenced by an intense staining of C3 with absence or low presence of immunoglobulins and components of the classical complement pathway in the immunofluorescence pattern of a kidney biopsy [3]. This form of nephritis can be detected in individuals with defects in the complement activation that is induced by circulating factors or the presence of rare gene variants in complement components of the alternative pathway.

In a recent classification proposed by Cook and Pickering [4], C3 GP was shown to be principally composed of three major subgroups (Figure 1): (i) *DDD* is characterized by intramembranous glomerular deposits of dense osmophilic material; (ii) *C3 glomerulonephritis* (C3 GN) is based on the presence of less dense deposits of C3 in the mesangial, subendothelial, and subepithelial areas of glomeruli; it also appears with the presence of circulating auto-antibodies against C3bBb, factor B (FB), and factor H (FH); (iii) *complement factor H-related 5 glomerulopathy* (CFHR5 GP) is caused by genetic variants of CFHR5. Differences in these three nephritides are based on the interpretation of data obtained by light microscopy, immunofluorescence/immunohistochemistry and EM, laboratory complement findings, and clinical data. However, in some cases, there is an overlap of clinical data and laboratory findings, suggesting the possibility of a disease continuum based on the dysregulation of the complement alternative pathway; this would be caused by acquired factors (autoantibodies) or genetic variants of some complement components of the alternative pathway.

## 2. Pathogenesis

The complement system is the first cornerstone of innate immunity, and in the presence of various infections, it induces the lysis of agents through the generation of the membrane attack complex (MAC) [5]. Moreover, the system modulates adaptive immunity.

**Figure 2:**
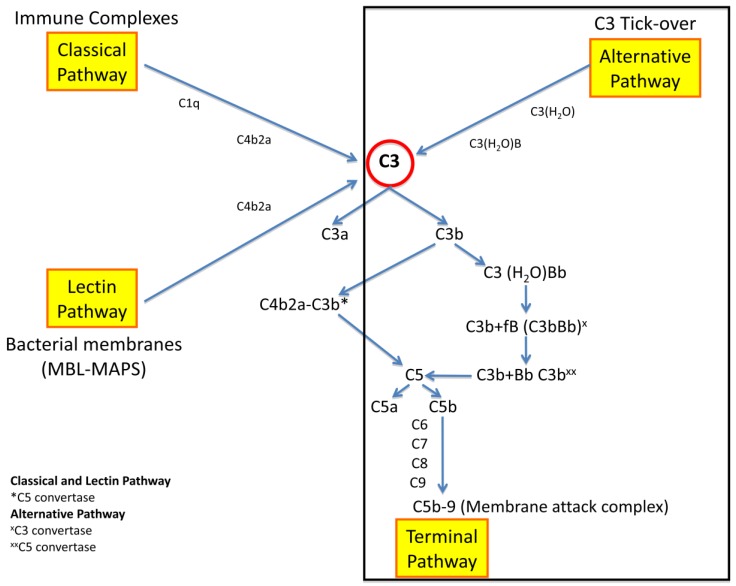
Complement system pathways.

The complement system can be activated through three different pathways, as illustrated in Figure 2.

The *classical pathway* is activated by circulating immune complexes, whereas the *lectin pathway* is activated by bacteria or their membrane fragments. Both pathways cleave C3 into C3a and C3b. C3a is an anaphylatoxin with a proinflammatory effect, whereas C3b binds a fragment of factor B (Bb), thus forming the C3 convertase (C3bBb). Additional production of C3b promotes the formation of the complex C3bBbC3b (C5 convertase), which cleaves C5 into C5a and C5b and combines with C6, C7, C8, and C9, thus forming the membrane attack complex (C5b-9) that induces the lysis of cellular membranes and the glomerular basement membrane (GBM).

The *alternative pathway* is continuously activated by the C3 tick-over at a low rate with the constant generation of C3b, which here is rapidly degraded. In this physiological process, C3 is hydrolyzed to C3(H_2_O) and combines with fB the complex C3(H_2_O)B. Then, this complex cleaves C3, generating C3b, which combines with Bb and forms the C3 convertase of the alternative pathway (C3bBb). In the presence of further C3b, the formed C5 convertase (C3bBbC3b) activates C5 with the sequential induction of the *terminal complement pathway* (C5b-9).

The three pathways of the complement system are modulated by proteins that regulate the system in the blood (fluid phase) and on the surface of cells (surface phase). In the fluid phase, the C1-inhibitor (C1-INH) downregulates the classical and lectin pathways; the C4 binding protein (C4bp) downregulates the classical pathway; clusterin and vimentin regulate C5b-9. The regulators of the complement in the surface phase system are the membrane cofactor protein (MCP, named CD46), CD59 that is a regulator of MAC formation, the decay accelerating factor (DAF, named CD55), and the complement receptor 1 (CR1).

**Figure 3:**
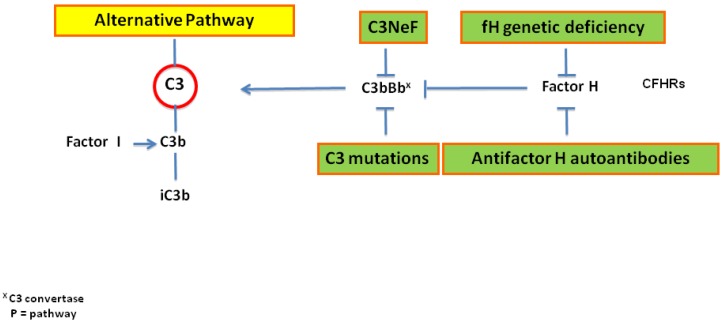
Schematic dysregulation of the C3 activation in C3 GP.

The alternative pathway is regulated by properdin, FB, FI, FH, and FH-related proteins. Properdin enhances the formation of C3 convertase and stabilizes it; thus, properdin prevents the action of FH. FH is the principal regulator of the alternative pathway both in the fluid phase and on the cellular surface; it inhibits the binding of C3b with FB, enhances C3 convertase dissociation, and the activity of FI, which cleaves C3b into inactivated C3b (iC3b). A dysregulated control of C3 convertase (C3bBb) can be caused by the abnormal activity of FH, as illustrated in Figure 3. The regulatory activity of this factor can be inhibited by the presence of genetic FH deficiency or autoantibodies against FH, which have been found in patients with C3 GP [6,7,8,9].

The activity of FH is regulated by the group of CFHRs that activate the alternative pathway by binding C3b and the activity of C3bBb convertase. CFHRs also compete with FH when attempting to bind to C3b deposited on the cellular surfaces. Therefore, the competition between FH and CFHRs influences the degree of C3b by inhibiting (predominant FH binding) or allowing (predominant FHR binding) to this process. The CFHR system is composed of five proteins that are coded by genes (CFHR1 to CFHR5) located near the CFH gene. CFHR genes may have rearrangements because the deletion or duplication of the first two consensus repeats with the presence of altered activity of CFHR proteins. Abnormal rearrangements include intragenic duplication in CFHR1 and CFHR5 and the presence of hybrid CFHR2-CFHR5 and CFHR3-CFHR1 genes. A deletion of the CFHR1 gene is associated with the abnormal activity of FH and reduced control of the activation of the alternative complement pathway in the circulation system. 

CFHR5 interacting with CFHR1 and CFHR2 may generate abnormal CFHR proteins that impact the function of C3 and/or C5 convertases in the fluid phase or on the glomeruli, causing C3 GP. More than 20 different mutations have been identified in the CFH-CFHR gene family of patients affected by C3 GP, as reported by Xiao et al. [10] and Merinero et al. [11]. A recent review on the process of CFH deregulation that is responsible for the C3 activation has been published by Barbour et al. [12].

An excessive activation of the alternative pathway may be caused by the presence of autoantibodies against the C3 convertase of the alternative pathway (C3NeF) and C3 convertase of the classical pathway (C4NeF) (Figure 3). In 1969, NeF was detected for the first time in a case of MPGN with persistent hypocomplementemia [13]. The serum of this patient was able to break down into C3 when it was incubated with normal human serum. The activity of NeF was attributed to the stabilization of the alternative complement pathway convertase (C3bBb), and it was named C3NeF [13,14]. Then, further studies [15,16,17,18] described various types of NeF stabilizing different convertases, thus preventing their cleavage and causing continuous activation of the complement system. Marinozzi et al. [19] detected a C3NeF that stabilizes the C5 convertase of the alternative pathway (C5NeF). C4NeF was first detected in a case of patients with postinfection glomerulonephritis and who had persistent low levels of C3 and C5 [20], and it was also discovered in a case of MPGN. C3NeF and C4NeF may coexist in the same patient [21], but plasma C3, consumption, and disease severity do not always correlate with the presence and activity of C3NeF in individuals with MPGN [22]. Other autoantibodies related to complement components, such as FB, FH, and C3b have been detected in a smaller percentage of patients [23]. A recent review regarding the use of diagnostic tools to detect and characterize NeFs was published by Donadelli et al. [24].

In conclusion, various autoantibodies enhance the activity of the alternative pathway in different ways. C3NeF binds and stabilizes the C3 convertase in the presence of properdin. C4NeF binds and stabilizes C3 convertase (C4bC2a) of the classical pathway, thus causing more production of C3b and C3bBb. Autoantibodies against factor H eliminate the regulatory function of this factor [25], whereas autoantibodies against factor B stabilize the enhancing function of factor B [26,27]. The presence of one of these autoantibodies is responsible for the development of C3 GP caused by an abnormal control of alternative complement pathway activation with reduced C3 degradation and increased C3 fragment deposition in the GBM.

## 3. Epidemiology 

C3 GP is a rare disease, so its frequency can only be approximately calculated. Cohort studies and an analysis of data from C3 GP registry have estimated an annual incidence of biopsy-proven DDD and C3 GN of one to two cases per million, with both sexes being affected equally [28]. The prevalence has been calculated as less than five cases per 1,000,000 in the United States and ~0.2–1.0 cases per 1,000,000 among the European population [25,29].

Instead, for CFHR5 GP, which was first observed in patients from Cyprus, data of incidences are lacking, while the prevalence value has been estimated at 140 cases per 1,000,000 Cypriot individuals [30].

## 4. Clinical Manifestations

Because of the low frequency of C3 GP, patient cohort studies are important and can provide valuable information regarding clinical presentation, pathological and laboratory features, and their correlation with disease outcomes. 

Table 1 shows the clinical and laboratory data of patients affected by C3 GP in different cohorts, as described by many investigators [23,25,29,31,32,33,34,35,36]. C3 GP involves mainly children and young people and may develop after an infectious episode (i.e., streptococcal infection) of the upper respiratory tract. However, the presence of rare variants linked to C3 and other components of the alternative pathway predisposes an individual to C3 GP susceptibility. 

The onset of the disease is characterized by micro- or macroscopic hematuria, light or heavy proteinuria, and a progressive reduction of renal function. Microhematuria is present in 90% of individuals, and episodes of gross hematuria may occur in 20% of patients in concomitance with upper respiratory tract infections. At the beginning of the disease and mainly in adults, light proteinuria may be present, which then becomes heavy over the course of the disease. The impairment of renal function is more frequent in adults and elderly subjects.

These clinical manifestations are common in all subtypes of C3 GP based on the aberrant control of the alternative complement pathway and sequential deposition of C3 in glomeruli. Therefore, renal biopsy remains the main approach for diagnosis. 

In **DDD**, the onset is characterized by nephrotic syndrome in 50% of the patients and acute nephritic syndrome in the other 50%. Hypertension may be present at the onset of the disease or detected during the course of the disease. These clinical manifestations are preceded by an acute episode of upper respiratory tract infection.

In a few cases, DDD shows at the fundus oculi drusen bodies that are electron-dense deposits of complement factors between the Bruch’s membrane and the retinal epithelium. This abnormality may be present at all ages and comes with a modest risk of progressive visual loss.

In some cases, patients with DDD may show acquired partial lipodistrophy that is characterized by the absence of subcutaneous fat in the face, but can also be present in the arms and upper portions of the trunk. However, partial lipodistrophy is more frequent in MPGN.

The outcome of DDD patients is poor, given that 50% of individuals progress to end-stage kidney disease (ESKD) within 10 years of diagnosis.

In **C3 GN**, the onset of the disease is characterized by microhematuria associated with hypertension in one-third of patients and nephritic syndrome in another third. The percentage of patients with ESKD is similar to that of patients with DDD. The disease involves mainly children and young adults. Microhematuria is present in 90% of individuals, and episodes of gross hematuria may occur in 20% of patients, along with upper respiratory tract infections. At the beginning of the disease light proteinuria that becomes heavy during the course of the disease is present but mainly in adults. The impairment of renal function is more frequent in adults.

**CFHR5 glomerulopathy** (CHFR5 GP) has been described in family studies; here, the abnormal activity of the complement factor H-related (CFHR) proteins or deregulation of CFH caused by CFH gene polymorphisms segregates in some members of the family [33,37,38]. Individuals with these genomic abnormalities show persistent microscopic hematuria that may be associated with proteinuria. The development of chronic renal failure progressing to ESKD is more frequent in men with proteinuria. In other patients, the usual presentation may be macroscopic hematuria in the concomitance of upper respiratory tract infection, as in IgA nephropathy, which mainly occurs in children.

However, familial cases of C3 GP have been observed in the presence of CFHR3-1 protein abnormalities [39] or the internal duplication of CFHR1 [40] or CFHR5, specifically in members of a Cypriot family [41].

## 5. Renal Biopsy

A renal biopsy is essential to identify C3 GP because it is the correct approach for distinguishing the different subtypes of MPGN.

### 5.1. Immunofluorescence Microscopy

The diagnosis of C3 GP is based on an immunofluorescence (IF) technique, which reveals C3 dominant staining, with C3 being the only positive immunoreactant or with others (IgG, IgA, IgM, C1q) but C3 at least 2 + stronger [42].

**Figure 4:**
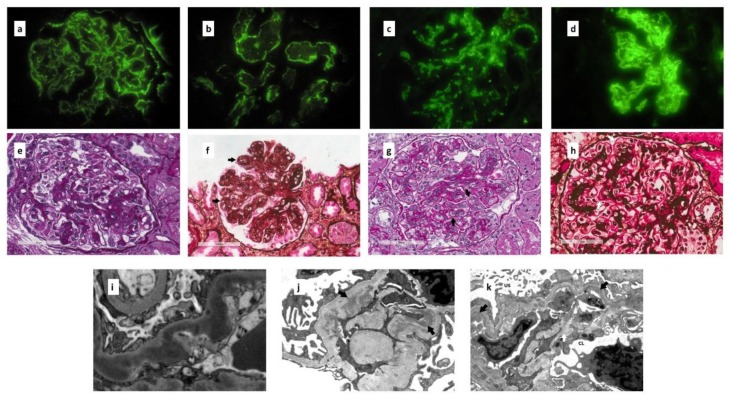
(**a**) Ribbon like pattern of C3 deposition along glomerular basement membrane in a patient with DDD (×400). (**b**) Linear C3 deposition along tubular basement membrane in DDD (×400). (**c**) Granular mesangial C3 deposits in a patient with a mesangial proliferative C3 GN (×400). (**d**) Coarsely granular mesangial and glomerular capillary wall C3 deposits in a patient with membranoproliferative pattern of C3 GN (×400). (**e**) Glomerulus with segmental mesangial proliferation in a patient with C3 GN (PAS). (**f**) Glomerulus with global membranoproliferative pattern of injury in a patient with C3 GN. Several aspects of glomerular basement membrane double contours (black arrows) are present (Jones silver stain). (**g**) Glomerulus with thickened strongly PAS positive glomerular basement membranes with several double contours (black arrows) (PAS) in a patient with DDD. (**h**) Silver negative glomerular basement membrane in a patient with DDD (white arrows). Glomerular basement membranes appear eosinophilic and refractile (Jones silver stain). (**i**) Highly osmiophilic electron dense deposits permeating lamina densa in a patient with DDD (TEM, ×8900). (**j**) Mesangial electron dense deposits (black arrows) in a patient with mesangial proliferative C3 GN (TEM, ×11000). (**k**) Subendothelial electron dense deposits (black arrows) with newly formed lamina densa (i.e., double contours) and cellular interposition (white arrow) in a patient with membranoproliferative patter of C3 GN (US: urinary space, CL: capillary lumen) (TEM, ×8900).

In DDD, C3 deposits are arranged in a ribbon-like (“garland”) pattern in the glomerular basement membranes with sometimes ring-shaped mesangial deposits (Figure 4a). Broad linear tubular basement membranes (~60%) (Figure 4b) and Bowman capsule deposits (~30%) can also be present in DDD.

In C3 GN, prominent granular C3 deposits are seen in the mesangium and/or glomerular basement membranes (Figure 4c,d). 

The absence of C4d in the kidney biopsy from proliferative GN has been found to be helpful, as noted by Sethi et al. [43], in distinguishing C3 GP from Ig complex-mediated and postinfection glomerulonephritis. Although helpful, this feature has been questioned by other studies [44].

C3 GP has now been included among the possible manifestations of monoclonal gammopathy of renal significance (MGRS) because 30–50% of patients >50 years of age with C3 GP have a detectable monoclonal Ig [45]. For this reason, careful examination of a renal biopsy and searching for a monoclonal Ig or light chain is mandatory. Failing to detect a monoclonal protein using an immunofluorescence technique from fresh frozen tissue should prompt reprocessing to formalin-fixed-paraffin-embedded tissue after pronase digestion. Actually, 10–20% of patients with a serum monoclonal component and finding of C3 GP on immunofluorescence have a proliferative GN with masked monoclonal deposits [45]. 

It is important to distinguish C3 deposition in C3 GN from other forms of glomerulonephritis, such as postinfection glomerulonephritis, in which immunoglobulin deposition occurs in the first phase of the disease and then disappears. In this case, persistence of heavy proteinuria and/or double contours on light microscopy (LM) may suggest the occurrence of C3 GP.

### 5.2. Light Microscopy

LM can vary greatly, presenting with different morphologic patterns. Mesangial proliferative glomerulonephritis (Figure 4e) and membranoproliferative glomerulonephritis (Figure 4f) are the most common patterns found, respectively, 33.3% and 50.8% of a cohort of 114 patients with C3 GP from the Mayo Clinic [23]. The membrane proliferative pattern in light microscopy is not different from that seen in idiopathic forms with Ig deposits. It is characterized by the thickening of glomerular basement membranes with double contours and cellular interposition. The features of exudative glomerulonephritis with endocapillary hypercellularity with or without polymorphonucleates are observed in 10–20% of cases. Extracellular proliferation with a crescent formation can also be present. In chronic phases of injury focal, segmental and/or global glomerulosclerosis and a fibrous crescent can be found. Fibrinoid necrosis is uncommon. 

In DDD, glomerular basement membranes appear eosinophilic and refractile and stain brightly with periodic acid-Schiff (PAS) and poorly with a Jones silver stain (Figure 4g,h).

### 5.3. Electron Microscopy

EM studies have refined the G3 GP classification into further categories (DDD and C3 GN) based on the appearance of deposits.

In DDD (formerly MPGN type II), EM shows highly osmiophilic dense deposits in the lamina densa of the glomerular basement membranes, resulting in an electron-dense appearance (Figure 4i). These deposits can also be found in the mesangium, Bowman capsule, tubular basement membranes, and small vessel walls.

In C3 GN, the “usual” electron-dense deposits are found in various locations: the mesangial (Figure 4j), subendothelial (Figure 4k), and intramembranous areas. Subepithelial humps can be found both in DDD and C3 GN.

## 6. Diagnosis

A single algorithm cannot define the diagnostic approach to C3 GP, which is based on the interpretation of individual cases through the integration of the information provided by the renal biopsy together with clinical, serological, and genetic assessments.

Because the clinical presentation of C3 GP in its different forms may vary from asymptomatic hematuria to nephrotic syndrome and acute kidney injury, a diagnosis of C3 GP should be taken into consideration virtually in all patients presenting features of glomerulonephritis (proteinuria, hematuria, renal failure, or active urine sediment).

**Figure 5:**
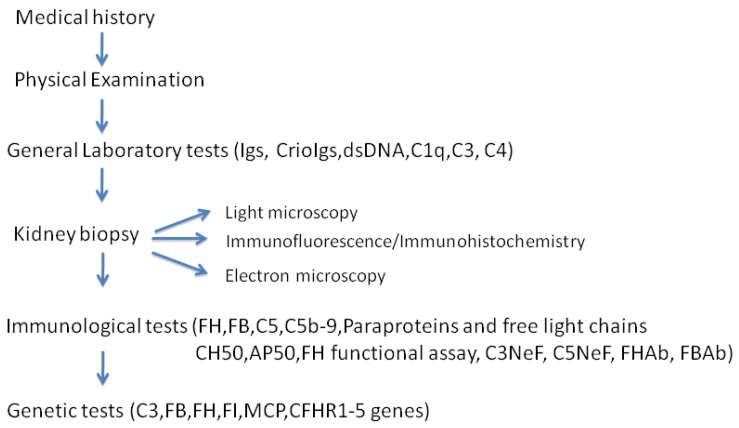
Clinical and Laboratory Work-up for diagnosis of C3 glomerulopathy.

The clinical and laboratory work-up for the diagnosis of C3 GP is illustrated in Figure 5. After collecting the medical history and providing an accurate examination of the physical status, the patient usually undergoes general laboratory evaluation, looking at immunity (measurement of the serum levels of immunoglobulins and detection of serum cryoglobulins) and the complement system (hemolytic assay of the classical and alternative pathway). In the presence of the specific activation of the alternative complement pathway (i.e., reduced C3 and normal C4 serum levels) C3 GP is highly suspected although not yet determined. The diagnosis of C3 GP is exclusively made based on renal histological findings. In particular, as previously shown, LM is not specific enough for C3 GP, but IF and EM are necessary to make a diagnosis of C3 GP and to distinguish between C3 GN and DDD.

C3-dominant glomerulonephritis is defined as C3 deposition in renal tissue of at least two orders of magnitude greater than that for any other immunoreactant (including Ig, C4, C1q, etc.). However, even when a C3 prevailing deposition is found, only a suspected diagnosis of C3 GP may be formulated, while the definitive diagnosis might require an ultra-structural analysis by EM.

Once the histological diagnosis of C3 GP has been realized, additional laboratory tests should be done to evaluate the serum levels of other components of the complement system, such as FH, FB, C5, and other complement regulatory proteins. Then, the work-up proceeds to a differential diagnosis and excludes secondary forms of glomerulonephritis. Investigations of the medical history should include a possible familial story of glomerulonephritis, unclear renal insufficiency, prior episodes of hematuria and/or proteinuria, and secondary causes, such as infections, autoimmune diseases, and paraproteinemia. Particular attention should be paid to recent infective episodes that may represent a trigger for complement activation. Moreover, all patients (especially if the age is more than 50 years) should be screened for the presence of paraproteins by serum electrophoresis-immunofixation and serum-free light chain measurement. Indeed, it has been shown that monoclonal gammopathy may be common in patients with a histological diagnosis of C3 GP [46]. If paraproteins are present, the patient should be referred to hematology consultation because in these cases, a clone-guided treatment might improve renal outcomes.

If secondary forms of complement activation are unlikely or excluded, all patients with C3 GP should undergo a comprehensive evaluation of the complement system, which includes measurements of the complement hemolytic activity, screening for circulating autoantibodies and genetic studies, which can help in defining the diagnosis and choosing the therapeutic approach [47].

The evaluation of the overall complement activity may be performed by evaluating the total complement hemolytic activity (CH50), the complement alternative pathway activity (AP50), or the complement FH functional activity [48]. Although functional tests may be considered the gold standard for studying the complement activity, they may actually have a significant rate of false-negative results, and their use still needs to be validated.

Moving to the specific details, serological studies of the complement AP should include the quantification of individual complement components, here bearing in mind that the results of these studies may change over the course of the disease. The most important complement components to evaluate are C3 and C4. Low C3 is found in a substantial number of patients with C3 GN (40–75%) and DDD (60%), while serum C4 levels are usually normal [23]. Moreover, serum FB, FH, FI, properdin, and membrane cofactor proteins (MCP or CD46) should also be tested, considering that a deficiency of these factors, often accompanied by low C3 levels, can be associated with altered AP activation [49]. However, complement activation may also be detected by measuring the decay products of complement activation; when elevated, they may reflect an increased turnover of C3 (C3a, C3d), C5 (C5a), and FB (Bb) [50]. Finally, considering that the activation of all complement pathways leads to the generation of the membrane attack complex (C5b-9), soluble levels of C5b-9 should be evaluated as an additional marker of complement activation [49]. Interestingly, it should be noted that small differences in complement biomarkers between C3 GN and DDD exist. For example, in C3 GN, the plasma properdin reduction and soluble C5b-9 elevation tend to be higher than in DDD. Thus, a complement component evaluation may also provide an indication of the disease in the differential diagnosis between C3GN and DDD.

Serological studies in patients with C3 GP should also include screening for autoantibodies, which may constitute the acquired causes of C3 GP. C3NeF and C5NeF are the most frequently identified autoantibodies (Figure 5). Various diagnostic tools can be used to detect NeFs [51]. Three of these tools are functional and hemolytic assays. The first method consists of the measurement of C3 fragments in the serum’s patient by two-dimensional immunoelectrophoresis, immunofixation electrophoresis, or Western blotting. The second test is based on a hemolytic assay that measures the lysis of sheep or rabbits erythrocytes after incubation with the serum or plasma of the patient. Finally, the presence of NeFs can be detected through the quantification of C3a and C5a anaphylotoxins. NeFs can be measured by binding assays that detect C3NeF by various ELISA techniques. Actually, they encompass a heterogeneous group of IgG and IgM autoantibodies that stabilize the alternative pathway of C3 and C5 convertase (C3bBb and C3bBbC3b, respectively), prolonging their survival and promoting massive C3 or C5 consumption [51]. C3NeF is found in 80% of patients affected by DDD and in 50% of patients affected by C3 GN. Detecting C3NeF can be done in many different ways, including ELISA, C3 convertase surface assay, and electrophoresis [52]. In addition, C4 nephritic factor (C4NeF), which stabilizes the C3 convertase of the classical and lectin pathways, has been identified in a small subset of patients with C3 GP, even if its role remains unclear [53]. Antifactor H antibodies are another group of auto-antibodies described in patients affected by C3 GP; they may be detected using an enzyme-linked immunosorbent assay and have been related to the presence of monoclonal gammopathy [54]. Finally, anti-FB antibodies have rarely been described, but their detection remains confined to research laboratories [26].

Because genetic factors have been reported in cohorts of patients with C3 GP, genetic testing should be considered in these patients [32] (Figure 5). In particular, genetic screening should include the evaluation of the C3, FB, FH, MCP, CFHR1-5, and FI genes to detect single nucleotide changes, small insertions and deletions, or more complex rearrangements, such as copy number variations [34]. However, the interpretation of a genetic analysis in C3 GP is often difficult for several reasons. First, genetic defects have been identified only in a small number of patients with C3 GP. Second, the actual pathogenetic and functional meaning of many mutations is still not defined. Finally, most genetic variants have a low penetrance, and combined variants may coexist, which also involves other genes, such as thrombomodulin (THBD) and diacylglycerol kinase-epsilon (DGKE) [55]. Thus, it is necessary to cautiously analyze the detected gene alterations to determine if they are specifically disease associated.

A different situation is found in the familial forms of C3 GP, where disease-specific gene mutations have been characterized. This is the case of patients affected by CFHR5 GP and who are of Cypriot origin, in which an internal duplication in the CFHR5 gene has been described as causative [37]. Interestingly, other forms of familial C3 GP have been related to rearrangements of the CFHR2-CFHR5 hybrid gene and abnormalities in CFHR1 and CFHR5 [39,40], suggesting that the CFHR1-5 gene family should be carefully assessed in all C3 GP cases. A detailed description of the methods to quantify the CFHR proteins in the serum and detect circulating autoantibodies has recently been published by Sanchez-Corral et al. [56].

In any case, although the precise value of genetic testing remains to be defined in a general clinical setting for C3 GP, a genetic cause of the disease should be taken into consideration, and genetic screening should be offered to all the family members of affected patients who carry a potentially causative mutation.

## 7. Differential Diagnosis

A differential diagnosis should be done in the presence of diagnosed C3 GP on either a native or transplanted kidney. In this case, it is necessary to investigate the concomitant presence of low plasma levels of C4, occurrence of C4NeF (C4bC2a), and paraproteins (i.e., monocolonal gammopathy) because in this case, the patient is affected by MPGN type 1.

C3 GP must be differentiated from other types of glomerulonephritides that have excessive activation of the C system in the presence of circulating immune complexes, causing postinfection glomerulonephritis or secondary glomerulonephritides (lupus nephritis, essential cryoglobulinemia, and others) [1]. Because the clinical presentation in these cases is similar, it is suggested that an accurate study of the complement system and genetic tests in specialized laboratories be carried out. However, a kidney biopsy is diriment in these patients because it shows a prevalent deposition of immunoglobulins, which is the characteristic pattern of these nephritides. In addition, postinfection glomerulonephritis should be differentiated from C3 GP when there is no spontaneous resolution of the disease within one to two months.

G3 GP can be diagnosed in the presence of false-negative staining for immunoglobulins by immunofluorescence on the frozen renal tissue sections of patients with essential cryoglobulinemia. In these cases, it is suggested to investigate the presence of C4 in a kidney biopsy to discover the potential occurrence of the low serum levels of circulating immune complexes or cryoglobulins.

In many cases, the prognosis of C3 GP is severe when it comes to the progression of renal damage to ESKD [28,32]. It is less severe in CFHR5 GP.

## 8. Recurrence or De Novo C3 GP after Kidney Transplatation

Patients with C3 GP are at high risk to develop the disease after kidney transplantation. Therefore, post-transplant monitoring and available optional therapies are necessary for improving the outcome of the patients. The recurrence is at high rate for both DDD and C3 GN. The recurrence of CHFR5 GP has been observed in the kidney allograft of patients, allowing for a family with CFHR gene abnormalities [57]. C3 GP occurs in over 50% of patients with native C3 GP [58]. The timing and clinical presentation may be different with DDD recurrence in later post-transplant time. The appearance of hematuria, proteinuria and reduction of eGFR is a strong indicator of C3 GP recurrence. However, LM, IF, and EM studies are necessary for a correct diagnosis. A recent report [59] suggests that aggressive renal lesions in native kidney and hybrid CFHR3 1 gene-related C3 GN contribute to more recurrence of the disease in the post-transplant time.

The approach to therapy has not been established because different treatments have been used in these patients. Recent data from Bomback et al. [60] in a prospective open-label, uncontrolled trial and by other investigators [61,62,63] suggest the administration of eculizumab which induces reduction of proteinuria and stabilization of the renal function. However, the heterogeneous treatment response suggests the opportunity to design a larger multicenter study in C3 GP patients after kidney transplantation. For this reason in 2017, a group of researchers across Europe and North and South America initiated the post-TrANsplant GlOmerular (TANGO) disease study, an observational multicenter cohort study with the aim to enroll patients who arrived to ESKD for different forms of glomerulonephritis, mainly C3 GP [64].

If eculizumab represents a successful therapy in atypical hemolytic uremic syndrome (AHUS) [65], the response rate is heterogeneous in patients with C3 GP [66]. The different response is due to the correct blockage of the terminal complement pathway in AHUS, whereas this therapy is questionable in patients with C3 GP because the disease is characterized by abnormal activation of the alternative complement pathway, abnormal cleavage of C3 and continuous deposition of C3 split products in glomeruli. This provides different response rate between the two diseases. 

Some considerations should be done on the use of anti-CD 20 (rituximab) that has been used in a small number of cases with different response to the treatment. This drug is correctly indicated in patients with kidney graft who develop MPGN with monoclonal IgG deposits caused by dysregulated B cells or plasma cells alone without hematologic malignancy [67]. However, sporadic use of rituximab in association with immunosuppressive drugs has shown failure in the treatment of many cases [62,68,69].

Recurrence or *de novo* C3 GP may receive other therapies that are under trough evaluation as (i) compostatin that is a C3 inhibitory peptide; (ii) CP40 is a compostatin analog [70]; (iii) monoclonal antibodies against C3b [71], FB [72] and properdin [73]. Moreover, soluble complement receptor 1(CR1)) may prevent the dysregulated alternative pathway caused by abnormal production of C3 convertase.

All these drugs can block the dysregulated alternative complement pathway induced by autoantibodies or genetic mutations.

## 9. Therapy

The current gap in the treatment of C3 GP derives from the absence of a robust understanding of its natural outcomes. Therefore, a specific treatment for different types of C3 GP has not been established. Recommendations have been obtained from case series and observational studies [12] because there are no randomized clinical trials for treatments. Current treatment is based on the inhibition of factors that activate the complement pathways, which is done by administering the available drugs as corticosteroids and antiproliferative drugs (cyclophoshamide, mycophenolate mofetil), monoclonal antibodies (rituximab), or complement inhibitors (eculizumab).

Children and adults with C3 GP in presence of mild or moderate proteinuria should receive supportive therapy with angiotensin-converting enzyme inhibitors or angiotensin receptor blockers. Patients with nephrotic syndrome and a decline in renal function should receive oral CYC or MMF plus a low dose of daily or alternate-day corticosteroids [33]. If despite this treatment nephrotic syndrome persists or the renal function impairs, tacrolimus or RTX can be considered although the percentage of its beneficial effect is very low [35,70].

Treatment with chemotherapeutic agents, immune-suppressive drugs, and complement inhibitors confer infection risks. Indeed, Koopman et al. [71] suggest vaccination prophylaxis against Streptococcus pneumonia, Neisseria meningitis, and Hemophilus influenzae.

In patients with C3 GN with abnormal deposition of C3 because of persistent activation of the alternative complement pathway, it is suggested that treatment with eculizumab (monoclonal anti-C5 antibody) may be administered. A prospective clinical trial in which six patients with native or post-transplant recurrent DDD or C3 GN received eculizumab therapy was the first clinical study to evaluate the benefit of this monoclonal antibody [60]. Other case reports described by Barbour et al. [12] have shown complete or partial remission of the clinical findings (significant reduction of serum creatinine and/or proteinuria). Recently, Le Quintrec et al. [36] evaluated a cohort of 26 patients (13 children/adolescents) with C3 GP who were treated with eculizumab. At the initiation of eculizumab, 40% of the patients had chronic kidney disease, while about 30% had a rapidly progressive disease. After eculizumab treatment, six (23%) patients achieved a complete clinical response, defined as a >50% decrease in serum creatinine and proteinuria, while six (23%) and 14 (54%) had partial or no response, respectively. Interestingly, at the time of diagnosis, patients who had a complete clinical response, when compared with others, presented a more severe disease associated with more extracapillary proliferation on kidney biopsy, suggesting that different response profiles to eculizumab treatment among C3 GP patients could be recognized. 

In individuals with monoclonal gammopathy that causes C3 GP (proteinuria and decline of renal function), a retrospective study of 50 patients with various types of monoclonal gammopathy [72] demonstrated that chemotherapy reduced proteinuria and improved renal function in 74% of patients, mainly in patients who had a reduction of gammopathy. Moreover, the hazard of ESRD was 83% lower in patients with reduced gammopathy. The same improvement was observed in other patients who received chemotherapy [23] in the presence of gammopathy because of lymphoproliferative disorders [73,74]. Many patients have shown an improvement in the clinical course, which is characterized by a reduction of proteinuria and improvement of renal function. However, the role of eculizumab remains to be defined. C3 GP remains the ideal disease in which anticomplement drugs (compostatin, monoclonal antibodies against C3, FB, properdin) can be tested [75,76,77,78].

Plasma exchange removes autoantibodies such as NeFs and mutated proteins, but it must be associated with immunosuppressive therapy for blocking antibody production. This approach has been used successfully in patients with CFH mutations [79,80,81]. The Consensus Report on C3 GP [2] concluded that an international pathology registry is necessary to define the different spectra of this disease and to divide patients into appropriate subgroups. Thus, the combination of well-defined clinical studies associated with a robust complement study would be ideal before enrolling patients in clinical trials. The final aim will be to treat homogenous cohorts before enrolling patients in clinical trials.

## 10. Conclusions

C3 GPs are orphan diseases of which the understanding of the pathophysiology of the complement system has improved laboratory and clinical diagnosis. However, a multidisciplinary approach that involves nephrologists, renal pathologists, and geneticists is helpful for a correct diagnosis and for choosing optimum therapeutic plans. More comprehensive genetic and biomarker studies are needed to refine our diagnostic approach. We should continue to improve the clinicopathological phenotype for designing correct multicenter trials that will give more information on the clinical course of the disease and the effect of new drugs. Treatment with a combination of immune-suppressive drugs or anticomplement agents can induce complete or partial remission in 50% of patients. Remission is more frequent in C3 GN than in DDD, whereas spontaneous remission is very rare. These diseases lead to ESKD in 50% of patients; some of them have persistent heavy proteinuria that is nonresponsive to therapy, ranging from ACEi-ARB to immunosuppressive drugs. Older age reduced renal function, and nephrotic syndrome at presentation are unfavorable prognostic factors. The recurrence of these diseases in patients after kidney transplantation is very frequent; therefore, post-transplant monitoring and available treatment options should be taken into consideration. Newer therapies such as monoclonal antibodies and recombinant proteins may be promising strategies in the near future.

## Figures and Tables

**Table 1:** Clinical and laboratory characteristics of patients affected by C3 GP enrolled in cohort studies.

Study Population						Clinical Findings			Histology	Complement Findings §				
Author-Year (Ref. No.)	Country	C3 GP Class	N	Age at Diagnosis (Years)	Male Sex (%)	Median SCr at Diagnosis (mg/dL)	Median UP at Diagnosis (g/24 h)	Main Clinical Presentation (%)	Main Histological Patterns (%)	Low C3 (%)	Low C4 (%)	C3Nef No. (%)	Complement Gene Variant Identified No. (%)	Other Complement Antibodies No. (%)
Athanasiou, 2011 [31]	Cyprus	CFHR5 GP	91	Range 12–88	47	N/A	N/A	H 90 Pu 38	MES	0	0	N/A	91/91 (100)	N/A
Servais, 2012 [32]	France	C3 GN	56	30	58.6	1.3	3.6	H 64 NS 27	MPGN (71), MES (29)	40	0	25 (45)	10 (18)	N/A
		DDD	29	12	43	1.6	5.6	H 76 NS 30		59	4	25 (86)	5 (17)	N/A
Mejeral-Thomas, 2014 [25]	UK/ Ireland	C3 GN	59	26	54	1.4	3	H 87 NS 44	MPGN (52), MES (24), Crescentic (5)	48	36	N/A	N/A	N/A
		DDD	21	12	43	0.9	3	H 86 NS 43	MPGN (62), MES (14), Crescentic (19)	79	15	N/A	N/A	N/A
Rabasco, 2015 [33]	Spain	C3 GN	60	27	57	1.4	3.8	NS 52 H 49	MPGN (75), MES (15)	63	N/A	11/23 (48)	3/23 (13)	0
Iatropoulos, 2016 [34]	Italy	C3 GN	52	14.6	42	N/A	N/A	H 86 NS 29	N/A	69	N/A	23/52 (44)	10/52 (20)	N/A
		DDD	21	15.9	38	N/A	N/A	H 90 NS29		86	N/A	16/21 (78)	3/21 (14)	N/A
Caliskan, 2017 [35]	Turkey	C3 GN (no subclass)	66	36	54	1.6	3.8	H 89 NS 48	MPGN (75), MES (24)	41	4	N/A	N/A	N/A
Bomback, 2018 [29]	USA	C3 GN	87	28.3	63.2	2	3.7	H 59 NS 33	MPGN (68.8), MES (17.5)	65	14	13/42 (31)	12/42 (28)	3/42 (7)
		DDD	24	40	67	2.1	4.4	H 82 NS 17	MPGN (45.8), MES (29.3)	64	14	1/9 (11)	3/9 (33)	1/9 (11)
Le Quintec, 2018 [36]	France	C3 GN (no subclass)	26	17.5	48	1	4.1	NS 73	MPGN (62)	88	N/A	16/24 (67)	5/22 (23)	2/22 (9)
Ravindran, 2018 [23]	USA *	C3 GN	102	41.5	59	1.6	2.5	H 87 Pu 38	MPGN (52), MES (34), Crescentic (7)	42	12	27/60 (46)	21/61 (39)	8/60 (13)
		DDD	12	31.5	25	1.4	6.5	H 92 Pu 75	MPGN (45), MES (27), Crescentic (16)	58	8	3/10 (30)	2/9 (22)	1/8 (12)

Abbreviations: C3G = C3 glomerulopathy; CeGN = C3 glomerulonephritis; DDD = dense deposition disease; CFHR5N = CFHR5 nephropathy; SCr = serum creatinine; UP = urinary protein; H = hematuria, Pu = proteinuria; NS = nephrotic syndrome; MPGN = Membrano-Proliferative Glomerulonephritis; MES = Mesangial expansion; C3Nef = C3 Nephritic factors; N/A = data not available from the study. Notes: * including 36 patients with Monoclonal immunoglobulin; § complement findings were often evaluated only in a part of the studied patients.

## References

[1] Schena F.P., Alpers G.E. (2015). Membranoproliferative glomerulonephritis and cryoglobulinemic glomerulonephritis. Comprehensive Clinical Nephrology.

[2] Pickering M.C., D’Agati V.D., Nester C.M., Smith R.J., Haas M., Appel G.B., Alpers C.E., Bajema I.M., Bedrosian C., Braun M. (2013). C3 glomerulopathy: Consensus report. Kidney Int..

[3] Fakhouri F., Frémeaux-Bacc V., Noël L.H., Cook H.T., Pickering M.C. (2010). C3 glomerulopathy: A new classification. Nat. Rev. Nephrol..

[4] Cook H.T., Pickering M.C. (2019). Clusters Not Classifications: Making Sense of Complement-Mediated Kidney Injury. J. Am. Soc. Nephrol..

[5] Ricklin D., Mastellos D.C., Reis E.S., Lambris J.D. (2018). The renaissance of complement therapeutics. Nat. Rev. Nephrol..

[6] Jokiranta T.S., Solomon A., Pangburn M.K., Zipfel P.F., Meri S. (1999). Nephritogenic lambda light chain dimer: A unique human miniautoantibody against complement factor H. J Immunol..

[7] Goodship T.H., Pappworth I.Y., Toth T., Denton M., Houlberg K., McCormick F., Warland D., Moore I., Hunze E.M., Staniforth S.J. (2012). Factor H autoantibodies in membranoproliferative glomerulonephritis. Mol. Immunol..

[8] Nozal P., Strobel S., Ibernon M., López D., Sánchez-Corral P., Rodríguez de Córdoba S., Józsi M., López-Trascasa M. (2012). Anti-factor H antibody affecting factor H cofactor activity in a patient with dense deposit disease. Clin. Kidney J..

[9] Blanc C., Togarsimalemath S.K., Chauvet S., Le Quintrec M., Moulin B., Buchler M., Jokiranta T.S., Roumenina L.T., Fremeaux-Bacchi V., Dragon-Durey M.A. (2015). Anti-factor H autoantibodies in C3 glomerulopathies and in atypical hemolytic uremic syndrome: One target, two diseases. J. Immunol..

[10] Xiao X., Pickering M.C., Smith R.J. (2014). C3 glomerulopathy: The genetic and clinical findings in dense deposit disease and C3 glomerulonephritis. Semin. Thromb. Hemost..

[11] Merinero H.M., García S.P., García-Fernández J., Arjona E., Tortajada A., Rodríguez de Córdoba S. (2018). Complete functional characterization of disease-associated genetic variants in the complement factor H gene. Kidney Int..

[12] Barbour T.D., Ruseva M.M., Pickering M.C. (2016). Update on C3 glomerulopathy. Nephrol. Dial. Transplant..

[13] Spitzer R.E., Vallota E.H., Forristal J., Sudora E., Stitzel A., Davis N.C., West C.D. (1969). Serum C’3 lytic system in patients with glomerulonephritis. Science.

[14] Arroyave C.M., Vallota E.H., Müller-Eberhard H.J. (1974). Lysis of human erythrocytes due to activation of the alternate complement pathway by nephritic factor (C3NeF). J. Immunol..

[15] Daha M.R., Fearon D.T., Austen K.F. (1976). Formation in the presence of C3 nephritic factor (C3NeF) of an alternative pathway C3 convertase containing uncleaved B. Immunology.

[16] Daha M.R., Austen K.F., Fearon D.T. (1977). The incorporation of C3 nephritic factor (C3NeF) into a stabilized C3 convertase, C3bBb (C3NeF), and its release after decay of convertase function. J. Immunol..

[17] Williams D.G., Bartlett A., Duffus P. (1978). Identification of nephritic factor as an immunoglobulin. Clin. Exp. Immunol..

[18] Józsi M., Reuter S., Nozal P., López-Trascasa M., Sánchez-Corral P., Prohászka Z., Uzonyi B. (2014). Autoantibodies to complement components in C3 glomerulopathy and atypical hemolytic uremic syndrome. Immunol. Lett..

[19] Marinozzi M.C., Chauvet S., Le Quintrec M., Mignotet M., Petitprez F., Legendre C., Cailliez M., Deschenes G., Fischbach M., Karras A. (2017). C5 nephritic factors drive the biological phenotype of C3 glomerulopathies. Kidney Int..

[20] Halbwachs L., Leveillé M., Lesavre P., Wattel S., Leibowitch J. (1980). Nephritic factor of the classical pathway of complement: Immunoglobulin G autoantibody directed against the classical pathway C3 convetase enzyme. J. Clin. Investig..

[21] Tanuma Y., Ohi H., Hatano M. (1990). Two types of C3 nephritic factor: Properdin-dependent C3NeF and properdin-independent C3NeF. Clin. Immunol. Immunopathol..

[22] Schena F.P., Pertosa G., Stanziale P., Vox E., Pecoraro C., Andreucci V.E. (1982). Biological significance of the C3 nephritic factor in membranoproliferative glomerulonephritis. Clin. Nephrol..

[23] Ravindran A., Fervenza F.C., Smith R.J.H., De Vriese A.S., Sethi S. (2018). C3 Glomerulopathy: Ten Years’ Experience at Mayo Clinic. Mayo Clin. Proc..

[24] Donadelli R., Pulieri P., Piras R., Iatropoulos P., Valoti E., Benigni A., Remuzzi G., Noris M. (2018). Unraveling the Molecular Mechanisms Underlying Complement Dysregulation by Nephritic Factors in C3G and IC-MPGN. Front. Immunol..

[25] Medjeral-Thomas N.R., O’Shaughnessy M.M., O’Regan J.A., Traynor C., Flanagan M., Wong L., Teoh C.W., Awan A., Waldron M., Cairns T. (2014). C3 glomerulopathy: Clinicopathologic features and predictors of outcome. Clin. J. Am. Soc. Nephrol..

[26] Strobel S., Zimmering M., Papp K., Prechl J., Józsi M. (2010). Anti-factor B autoantibody in dense deposit disease. Mol. Immunol..

[27] Chen Q., Müller D., Rudolph B., Hartmann A., Kuwertz-Bröking E., Wu K., Kirschfink M., Skerka C., Zipfel P.F. (2011). Combined C3b and factor B autoantibodies and MPGN type II. N Engl. J. Med..

[28] Smith R.J.H., Alexander J., Barlow P.N., Botto M., Cassavant T.L., Cook H.T., deCórdoba S.R., Hageman G.S., Jokiranta T.S., Kimberling W.J. (2007). Dense Deposit Disease Focus Group. New approaches to the treatment of dense deposit disease. J. Am. Soc. Nephrol..

[29] Bomback A.S., Santoriello D., Avasare R.S., Regunathan-Shenk R., Canetta P.A., Ahn W., Radhakrishnan J., Marasa M., Rosenstiel P.E., Herlitz L.C. (2018). C3 glomerulonephritis and dense deposit disease share a similar disease course in a large United States cohort of patients with C3 glomerulopathy. Kidney Int..

[30] Smith R.J.H., Appel G.B., Blom A.M., Cook H.T., D’Agati V.D., Fakhouri F., Fremeaux-Bacchi V., Józsi M., Kavanagh D., Lambris J.D. (2019). C3 glomerulopathy—Understanding a rare complement-driven renal disease. Nat. Rev. Nephrol..

[31] Athanasiou Y., Voskarides K., Gale D.P., Damianou L., Patsias C., Zavros M., Maxwell P.H., Cook H.T., Demosthenous P., Hadjisavvas A. (2011). Familial C3 glomerulopathy associated with CFHR5 mutations: Clinical characteristics of 91 patients in 16 pedigrees. Clin. J. Am. Soc. Nephrol..

[32] Servais A., Noël L.H., Roumenina L.T., Le Quintrec M., Ngo S., Dragon-Durey M.A., Macher M.A., Zuber J., Karras A., Provot F. (2012). Acquired and genetic complement abnormalities play a critical role in dense deposit disease and other C3 glomerulopathies. Kidney Int..

[33] Rabasco C., Cavero T., Román E., Rojas-Rivera J., Olea T., Espinosa M., Cabello V., Fernández-Juarez G., González F., Ávila A. (2015). Spanish Group for the Study of Glomerular Diseases (GLOSEN). Effectiveness of mycophenolate mofetil in C3 glomerulonephritis. Kidney Int..

[34] Iatropoulos P., Noris M., Mele C., Piras R., Valoti E., Bresin E., Curreri M., Mondo E., Zito A., Gamba S. (2016). Complement gene variants determine the risk of immunoglobulin-associated MPGN and C3 glomerulopathy and predict long-term renal outcome. Mol. Immunol..

[35] Caliskan Y., Torun E.S., Tiryaki T.O., Oruc A., Ozluk Y., Akgul S.U., Temurhan S., Oztop N., Kilicaslan I., Sever M.S. (2017). Immunosuppressive Treatment in C3 Glomerulopathy: Is it Really Effective?. Am. J. Nephrol..

[36] Le Quintrec M., Lapeyraque A.L., Lionet A., Sellier-Leclerc A.L., Delmas Y., Baudouin V., Daugas E., Decramer S., Tricot L., Cailliez M. (2018). Patterns of Clinical Response to Eculizumab in Patients with C3 Glomerulopathy. Am. J. Kidney Dis..

[37] Gale D.P., Goicoechea de Jorge E.G., Cook H.T., Martinez-Barricate R., Hadjisavvas A., McLean A.G. (2010). Identification of a mutation in complement factor H-related protein 5 in patients of Cypriot origin with glomerulonephritis. Lancet.

[38] Martínez-Barricarte R., Heurich M., Valdes-Cañedo F., Vazquez-Martul E., Torreira E., Montes T., Tortajada A., Pinto S., Lopez-Trascasa M., Morgan B.P. (2010). Human C3 mutation reveals a mechanism of dense deposit disease pathogenesis and provides insights into complement activation and regulation. J. Clin. Investig..

[39] Malik T.H., Lavin P.J., Goicoechea de Jorge E., Vernon K.A., Rose K.L., Patel M.P.A. (2012). A hybrid CFHR3-1 gene causes familial C3 glomerulopathy. J. Am. Soc. Nephrol..

[40] Tortajada A., Ye’benes H., Abarrategui-Garrido C., Anter J., García-Fernández J.M., Martínez-Barricarte R., Alba-Domínguez M., Malik T.H., Bedoya R., Cabrera Pérez R. (2013). C3 glomerulopathy associated CFHR1 mutation alters FHR oligomerization and complement regulation. J. Clin. Investig..

[41] Medjeral-Thomas N.R., Troldborg A., Constantinou N., Lomax-Browne H.J., Hansen A.G., Willicombe M., Pusey C.D., Cook H.T., Thiel S., Pickering M.C. (2017). Progressive IgA Nephropathy is Associated with low circulating mannan-binding lectin-associated serine protease-3 (MASP-3) and increased glomerular factor H-related protein-5 (FHR5) Deposition. Kidney Int. Rep..

[42] Hou J., Markowitz G.S., Bomback A.S., Appel G.B., Herlitz L.C., Barry Stokes M., D’Agati V.D. (2014). Toward a working definition of C3 glomerulopathy by immunofluorescence. Kidney Int..

[43] Sethi S., Nasr S.H., De Vriese A.S., Fervenza F.C. (2015). C4d as a diagnostic tool in proliferative GN. J. Am. Soc. Nephrol..

[44] Singh G., Singh S.K., Nalwa A., Lavleen S., Pradeep I., Barwad A., Sinha A., Hari P., Bagga A., Bagchi S. (2019). Glomerular C4d staining does not exclude a C3 Glomerulopathy. Kidney Int. Rep..

[45] Leung N., Bridoux F., Batuman V., Chaidos A., Cockwell P., D’Agati V.D., Dispenzieri A., Fervenza F.C., Fermand J.P., Gibbs S. (2019). The evaluation of monoclonal gammopathy of renal significance: A consensus report of the International Kidney and Monoclonal Gammopathy Research Group. Nat. Rev. Nephrol..

[46] Zand L., Kattah A., Fervenza F.C., Smith R.J., Nasr S.H., Zhang Y., Vrana J.A., Leung N., Cornell L.D., Sethi S. (2013). C3 glomerulonephritis associated with monoclonal gammopathy: A case series. Am. J. Kidney Dis..

[47] Angioi A., Fervenza F.C., Sethi S., Zhang Y., Smith R.J., Murray D., Van Praet J., Pani A., De Vriese A.S. (2016). Diagnosis of complement alternative pathway disorders. Kidney Int..

[48] Grumach A.S., Kirschfink M. (2014). Are complement deficiencies really rare? Overview on prevalence, clinical importance and modern diagnostic approach. Mol. Immunol..

[49] Zhang Y., Nester C.M., Martin B., Skjoedt M.O., Meyer N.C., Shao D., Borsa N., Palarasah Y., Smith R.J. (2014). Defining the complement biomarker profile of C3 glomerulopathy. Clin. J. Am. Soc. Nephrol..

[50] Fervenza F.C., Sethi S. (2014). Circulating complement levels and C3 glomerulopathy. Clin. J. Am. Soc. Nephrol..

[51] Corvillo F., Okrój M., Nozal P., Melgosa M., Sánchez-Corral P., López-Trascasa M. (2019). Nephritic Factors: An Overview of Classification, Diagnostic Tools and Clinical Associations. Front. Immunol..

[52] Zhang Y., Meyer N.C., Wang K., Nishimura C., Frees K., Jones M., Katz L.M., Sethi S., Smith R.J. (2012). Causes of alternative pathway dysregulation in dense deposit disease. Clin. J. Am. Soc. Nephrol..

[53] Ohi H., Yasugi T. (1994). Occurrence of C3 nephritic factor and C4 nephritic factor in membranoproliferative glomerulonephritis (MPGN). Clin. Exp. Immunol..

[54] Watson R., Lindner S., Bordereau P., Hunze E.M., Tak F., Ngo S., Zipfel P.F., Skerka C., Dragon-Durey M.A., Marchbank K.J. (2014). Standardisation of the factor H autoantibody assay. Immunobiology.

[55] Osborne A.J., Breno M., Borsa N.G., Bu F., Frémeaux-Bacchi V., Gale D.P., van den Heuvel L.P., Kavanagh D., Noris M., Pinto S. (2018). Statistical Validation of Rare Complement Variants Provides Insights into the Molecular Basis of Atypical Hemolytic Uremic Syndrome and C3 Glomerulopathy. J. Immunol..

[56] Sánchez-Corral P., Pouw R.B., López-Trascasa M., Józsi M. (2018). Self-Damage Caused by Dysregulation of the Complement Alternative Pathway: Relevance of the Factor H Protein Family. Front. Immunol..

[57] Vernon K.A., Gale D.P., de Jorge E.G., McLean A.G., Galliford J., Pierides A., Maxwell P.H., Taube D., Pickering M.C., Cook H.T. (2011). Recurrence of complement factor H-related protein 5 nephropathy in a renal transplant. Am. J. Transplant..

[58] Cochat P., Fargue S., Mestrallet G., Jungraithmayr T., Koch-Nogueira P., Ranchin B., Zimmerhackl L.B. (2009). Disease recurrence in paediatric renal transplantation. Pediatr. Nephrol..

[59] Wong L., Moran S., Lavin P.J., Dorman A.M., Conlon P.J. (2016). Kidney transplant outcomes in familial C3 glomerulopathy. Clin. Kidney J..

[60] Bomback A.S., Smith R.J., Barile G.R., Zhang Y., Heher E.C., Herlitz L., Stokes M.B., Markowitz G.S., D’Agati V.D., Canetta P.A. (2012). Eculizumab for dense deposit disease and C3 glomerulonephritis. Clin. J. Am. Soc. Nephrol..

[61] Abbas F., El Kossi M., Kim J.J., Shaheen I.S., Sharma A., Halawa A. (2018). Complement-mediated renal diseases after kidney transplantation—Current diagnostic and therapeutic options in de novo and recurrent diseases. World J. Transplant..

[62] Sánchez-Moreno A., De la Cerda F., Cabrera R., Fijo J., López-Trascasa M., Bedoya R., Rodríguez de Córdoba S., Ybot-González P. (2014). Eculizumab in dense-deposit disease after renal transplantation. Pediatr. Nephrol..

[63] Lim W.H., Shingde M., Wong G. (2019). Recurrent and de novo Glomerulonephritis after Kidney Transplantation. Front. Immunol..

[64] Uffing A., Pérez-Sáez M.J., La Manna G., Comai G., Fischman C., Farouk S., Manfro R.C., Bauer A.C., Lichtenfels B., Mansur J.B. (2018). A large, international study on post-transplant glomerular diseases: The TANGO project. BMC Nephrol..

[65] Legendre C.M., Licht C., Muus P., Greenbaum L.A., Babu S., Bedrosian C., Bingham C., Cohen D.J., Delmas Y., Douglas K. (2013). Terminal complement inhibitor eculizumab in atypical hemolytic-uremic syndrome. N. Engl. J. Med..

[66] Welte T., Arnold F., Kappes J., Seidl M., Häffner K., Bergmann C., Walz G., Neumann-Haefelin E. (2018). Treating C3 glomerulopathy with eculizumab. BMC Nephrol..

[67] Von Visger J., Cassol C., Nori U., Franco-Ahumada G., Nadasdy T., Satoskar A.A. (2019). Complete biopsy-proven resolution of deposits in recurrent proliferative glomerulonephritis with monoclonal IgG deposits (PGNMIGD) following rituximab treatment in renal allograft. BMC Nephrol..

[68] McCaughan J.A., O’Rourke D.M., Courtney A.E. (2012). Recurrent dense deposit disease after renal transplantation: An emerging role for complementary therapies. Am. J. Transplant..

[69] Zand L., Lorenz E.C., Cosio F.G., Fervenza F.C., Nasr S.H., Gandhi M.J., Smith R.J., Sethi S. (2014). Clinical findings, pathology, and outcomes of C3GN after kidney transplantation. J. Am. Soc. Nephrol..

[70] Rudnicki M. (2017). Rituximab for Treatment of Membranoproliferative Glomerulonephritis and C3 Glomerulopathies. BioMed Res. Int..

[71] Koopman J.J.E., Teng Y.K.O., Boon C.J.F., van den Heuvel L.P., Rabelink T.J., van Kooten C., de Vries A.P.J. (2019). Diagnosis and treatment of C3 glomerulopathy in a center of expertise. Neth. J. Med..

[72] Chauvet S., Roumenina L.T., Aucouturier P., Marinozzi M.C., Dragon-Durey M.A., Karras A., Delmas Y., Le Quintrec M., Guerrot D., Jourde-Chiche N. (2018). Both Monoclonal and Polyclonal Immunoglobulin Contingents Mediate Complement Activation in Monoclonal Gammopathy Associated-C3 Glomerulopathy. Front. Immunol..

[73] Chauvet S., Frémeaux-Bacchi V., Petitprez F., Karras A., Daniel L., Burtey S., Choukroun G., Delmas Y., Guerrot D., François A. (2017). Treatment of B-cell disorder improves renal outcome of patients with monoclonal gammopathy-associated C3 glomerulopathy. Blood.

[74] Sethi S., Sukov W.R., Zhang Y., Fervenza F.C., Lager D.J., Miller D.V., Cornell L.D., Krishnan S.G., Smith R.J. (2010). Dense deposit disease associated with monoclonal gammopathy of undetermined significance. Am. J. Kidney Dis..

[75] Zhang Y., Shao D., Ricklin D., Hilkin B.M., Nester C.M., Lambris J.D., Smith R.J. (2015). Compstatin analog Cp40 inhibits complement dysregulation in vitro in C3 glomerulopathy. Immunobiology.

[76] Paixão-Cavalcante D., Torreira E., Lindorfer M.A., Rodriguez de Cordoba S., Morgan B.P., Taylor R.P., Llorca O., Harris C.L. (2014). A humanized antibody that regulates the alternative pathway convertase: Potential for therapy of renal disease associated with nephritic factors. J. Immunol..

[77] Subías M., Tortajada A., Gastoldi S., Galbusera M., López-Perrote A., Lopez L.J., González-Fernández F.A., Villegas-Martínez A., Dominguez M., Llorca O. (2014). A novel antibody against human factor B that blocks formation of the C3bB proconvertase and inhibits complement activation in disease models. J. Immunol..

[78] Pauly D., Nagel B.M., Reinders J., Killian T., Wulf M., Ackerman S., Ehrenstein B., Zipfel P.F., Skerka C., Weber B.H. (2014). A novel antibody against human properdin inhibits the alternative complement system and specifically detects properdin from blood samples. PLoS ONE.

[79] Krmar R.T., Holtbäck U., Linné T., Berg U.B., Celsi G., Söderberg M.P., Wernerson A., Szakos A., Larsson S., Skattum L. (2011). Acute renal failure in dense deposit disease: Complete recovery after combination therapy with immunosuppressant and plasma exchange. Clin. Nephrol..

[80] Häffner K., Michelfelder S., Pohl M. (2015). Successful therapy of C3Nef-positive C3 glomerulopathy with plasma therapy and immunosuppression. Pediatr. Nephrol..

[81] Kurtz K.A., Schlueter A.J. (2002). Management of membranoproliferative glomerulonephritis type II with plasmapheresis. J. Clin. Apher..

